# Deep learning alignment of bidirectional raster scanning in high speed photoacoustic microscopy

**DOI:** 10.1038/s41598-022-20378-2

**Published:** 2022-09-28

**Authors:** Jongbeom Kim, Dongyoon Lee, Hyokyung Lim, Hyekyeong Yang, Jaewoo Kim, Jeesu Kim, Yeonggeun Kim, Hyung Ham Kim, Chulhong Kim

**Affiliations:** 1 Departments of Electrical Engineering, Mechanical Engineering, Convergence IT Engineering, Interdisciplinary Bioscience and Bioengineering, Medical Device Innovation Center, Graduate School of Artificial Intelligence, Pohang University of Science and Technology (POSTECH), 77 Cheongam-ro, Nam-gu, Pohang, Gyeongbuk 37673 Republic of Korea; 2 Department of Optics and Mechatronics Engineering, Pusan National University, Busan, 46241 Republic of Korea; 3 Opticho, 532, CHANGeUP GROUND, 87 Cheongam-ro, Nam-gu, Pohang, Gyeongsangbuk 37673 Republic of Korea; 4 Present Address: Samsung Electronics, 1, Samsungjeonja-ro, Hwaseong-si, Gyeonggi-do Republic of Korea

**Keywords:** Biomedical engineering, Imaging and sensing, Microscopy

## Abstract

Simultaneous point-by-point raster scanning of optical and acoustic beams has been widely adapted to high-speed photoacoustic microscopy (PAM) using a water-immersible microelectromechanical system or galvanometer scanner. However, when using high-speed water-immersible scanners, the two consecutively acquired bidirectional PAM images are misaligned with each other because of unstable performance, which causes a non-uniform time interval between scanning points. Therefore, only one unidirectionally acquired image is typically used; consequently, the imaging speed is reduced by half. Here, we demonstrate a scanning framework based on a deep neural network (DNN) to correct misaligned PAM images acquired via bidirectional raster scanning. The proposed method doubles the imaging speed compared to that of conventional methods by aligning nonlinear mismatched cross-sectional B-scan photoacoustic images during bidirectional raster scanning. Our DNN-assisted raster scanning framework can further potentially be applied to other raster scanning-based biomedical imaging tools, such as optical coherence tomography, ultrasound microscopy, and confocal microscopy.

## Introduction

Photoacoustic imaging (PAI) is a hybrid biomedical imaging technique that detects ultrasonic waves induced by the excitation of short optical pulses^1–7^. When excited by an optical pulse, biomolecules experience an increase in temperature due to optical absorption, and hence a pressure rise due to thermo-elastic expansion. The pressure rise results in the generation of ultrasonic waves called photoacoustic (PA) waves. Optical-resolution photoacoustic microscopy (OR-PAM) is a type of microscopic PAI that uses a tightly focused optical beam to detect emitted ultrasound in the optical ballistic or quasi-ballistic regime to achieve a high lateral resolution and signal-to-noise ratio (SNR) with a point-by-point raster scan^2^. OR-PAM has made significant contributions to the exploration of molecular, anatomical, and functional information for in vivo animal and human imaging in a variety of research fields, including biology, oncology, neurology, ophthalmology, dermatology, and pathology^2,8–20^.

Over the past few years, OR-PAM has utilized various high-speed scanning tools such as a voice-coil stage, microelectromechanical system (MEMS) scanners, polygonal mirror scanners, and galvanometer scanners to enhance the effective B-scan rate under point-by-point raster scan^2,8–15,21–24^. In particular, water-immersible MEMS and galvanometer scanners with tunable scanning ranges have been extensively used in various studies, accelerating the B-scan imaging speed up to hundreds of Hz^11–15,22,23^. This high scanning speed allows us to adapt a highly repeated pulsed laser system operating at several hundred kHz in OR-PAM, significantly shortening the imaging acquisition time. However, high-speed water-immersible scanners exhibit unstable raster scanning performance owing to heat and vibration. Owing to this instability, the time interval between scanning points are non-uniform, resulting in a misalignment between the bidirectional raster scanning paths. Consequently, the PA images are generally reconstructed using only unidirectionally collected data, while the data obtained from the other path are is discarded at the cost of doubling the image acquisition time.

Deep learning (DL), which is a rapidly developing field, has addressed the challenges associated with enhancement, translation, segmentation, classification, and medical decision-making in biomedical imaging tools such as PAI, computed tomography (CT), ultrasound imaging, magnetic resonance angiography (MRI), and optical microscopy^25–37^. Specifically, an interesting application of DL techniques lies in improving the imaging system performance, such as quality and speed^26,27,37,38^. Here, we present a scanning framework based on deep neural networks (DNNs) to halve the imaging acquisition time by correcting the misaligned OR-PAM images obtained via bidirectional scanning. Rather than using simulated or image-processed data, we trained and validated our DNN by using paired *in-vivo* OR-PAM images obtained via unidirectional and bidirectional scanning. This training strategy enabled us to enhance the bidirectional scanning images acquired in real imaging scenarios. Our DNN-based scanning approach to OR-PAM with raster scanning accelerates the image acquisition time, making it twice as fast, and it could contribute to the removal of artifacts from other raster scanning-based biomedical imaging tools, such as optical coherence tomography (OCT), ultrasound microscopy, and confocal microscopy.

## Results

### Optical-resolution photoacoustic microscopy (OR-PAM) system and scanning scenarios

**Figure 1:**
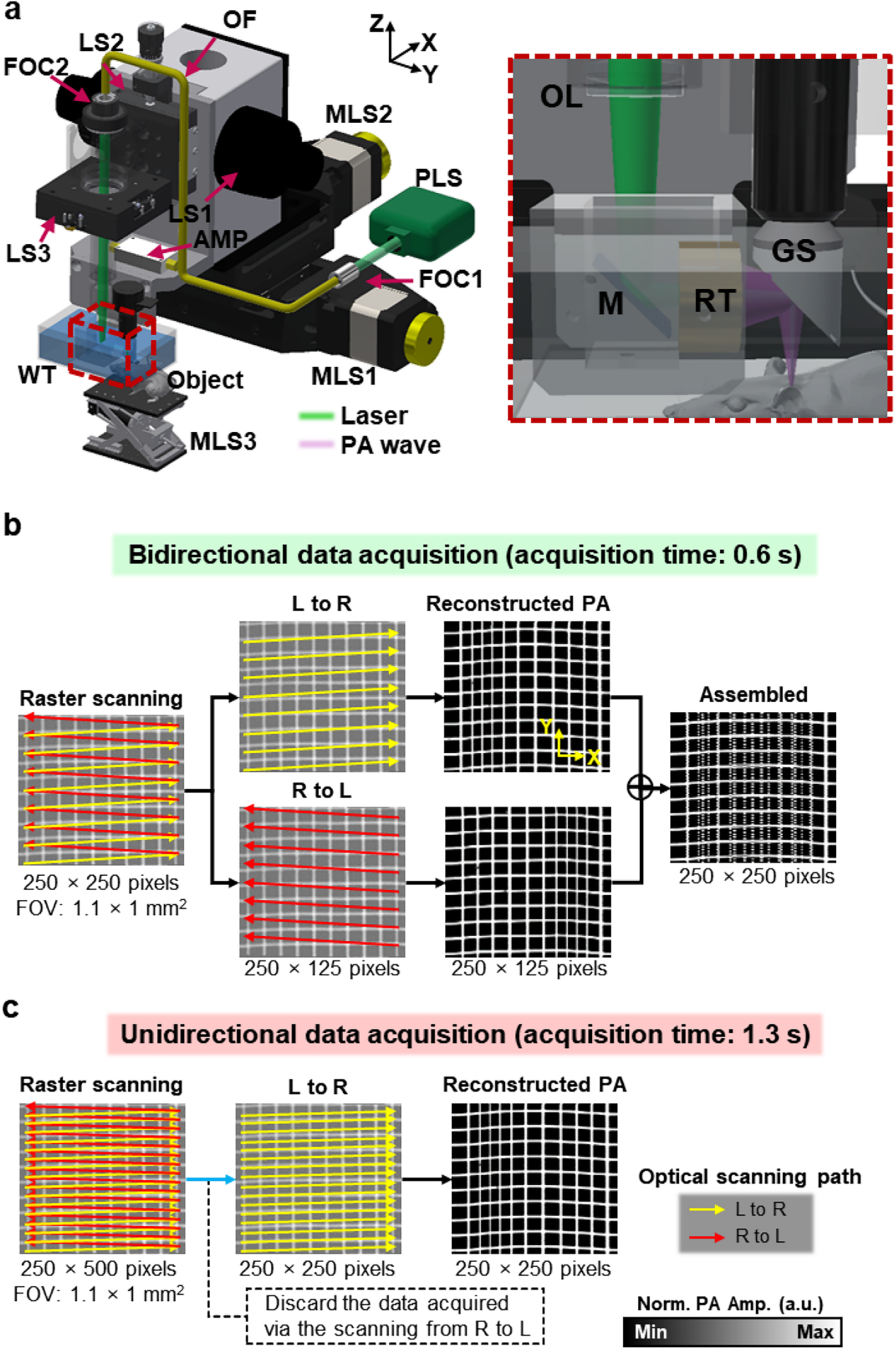
(**a**) Configuration of an optical-resolution photoacoustic microscopy (OR-PAM) system. Scanning and imaging acquisition scenarios with (**b**) bidirectional and (**c**) unidirectional raster scanning. OF, optical fiber; LS, linear stage; FOC, fiber optic collimator; MLS, motorized linear stage; PLS, pulsed laser system; AMP, amplifier; WT, water tank; OL, objective lens; RT, ring transducer; GS, galvanometer scanner; M, mirror; L, left; R, right; FOV, field of view; PA, photoacoustic; and Norm. PA Amp., normalized photoacoustic amplitude.

Figure 1 illustrates the configuration of the OR-PAM system and the difference between bidirectional and unidirectional raster scanning patterns. In general, high-speed OR-PAM systems adopt raster scanning methods that combine fast angular scanning using a water immersible opto-ultrasound scanner and slow linear scanning along the x- and y-axes, respectively^10–12^. Our OR-PAM system employed a water-immersible waterproof galvanometer scanner for fast angular scanning and a linear motorized scanner for slow linear scanning (Fig. 1a). As with the previously reported galvanometer OR-PAM, we mounted the galvanometer scanner so that only the mirror part was submersed^12^. This configuration enabled the scanner to direct both laser pulses and PA waves in the water without causing damage to the scanner. To achieve fast and high-contrast vascular imaging, a nanosecond pulsed laser with a maximum pulse repetition rate (PRR) of 600 kHz and optical wavelength of 532 nm was used. A laser beam from the system was collimated via a fiber optic collimator (FOC), delivered through an optical fiber, and recollimated via another FOC. The output optical beam from the optical fiber was then focused by an objective lens. The focused laser beam passed through a hole in the center of the ring-type ultrasound transducer and shone upon the sample. The generated PA wave was detected by the transducer, which was coaxially and confocally focused with light pulses to maximize the SNR measured in vivo to be 47.1 dB, which is superior to the previously reported result^12^. The OR-PAM system achieved a lateral resolution of 9.3 μm with a scanning range of ~ 1 mm and 1000 pixels, and an axial resolution of 117 μm with a scanning range of ~ 1 mm and 100 pixels (Supplementary Fig. S1). According to the theoretical calculations, the lateral resolution of 8.5 μm for the 0.032 optical numerical aperture and the axial resolution of 113 μm for a central ultrasonic frequency of 20 MHz and a − 6 dB acoustic bandwidth of 60% correspond to the achieved resolutions^1^.

Two methods, bidirectional and unidirectional, are used when acquiring data through raster scanning. Bidirectional data acquisition reconstructs images using all of the data obtained by scanning bidirectionally along the x-axis, which is an ideal case (Fig. 1b). However, in reality, the reconstructed PA images are distorted with the bidirectionally acquired data (Fig. 1b). Therefore, unidirectional data acquisition is preferable to bidirectional data acquisition because of the misalignment between bidirectionally obtained images (Fig. 1c). To create an image with the same number of pixels using unidirectional acquisition, the system is initially forced to scan bidirectionally and acquire twice as many pixels along one direction (e.g., 250 × 500 pixels in Fig. 1c). Data obtained from one direction is then discarded. In other words, even if the data is bidirectionally acquired (e.g., 250 × 500 pixels in Fig. 1c), only the data obtained in one direction is used when reconstructing an OR-PAM image (e.g., 250 × 250 pixels in Fig. 1c), which doubles the data acquisition time when compared to the method in Fig. 1b.

### Deep neural network (DNN) to correct misalignmented OR-PAM images with bidirectional raster scanning

**Figure 2:**
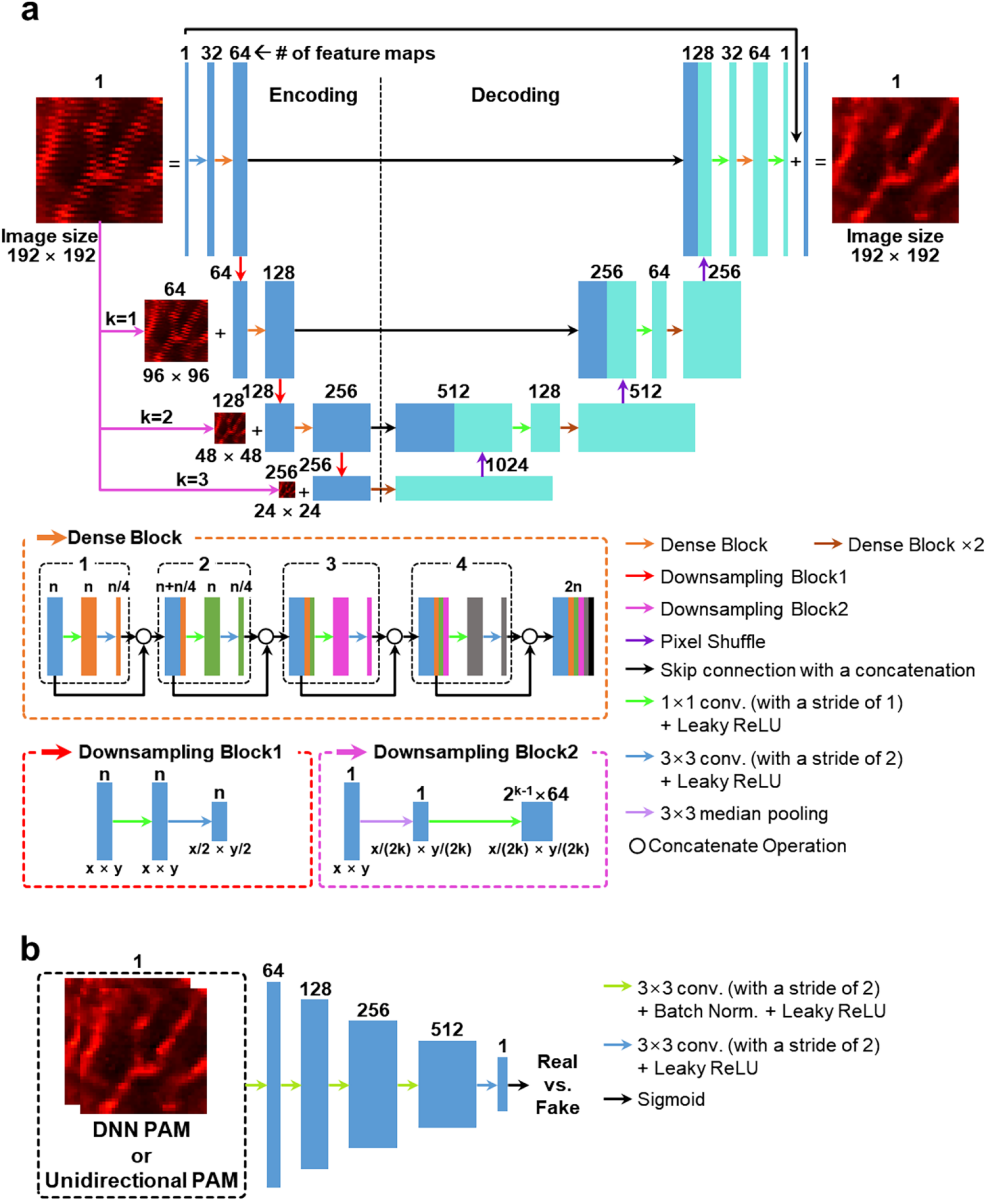
Visual representation of MS-FD-U-Net GAN. (**a**) Generator and (**b**) discriminator architectures of MS-FD-U-Net.

The bidirectionally obtained OR-PAM images were initially pre-processed. These pre-processed OR-PAM images were then fed as inputs to a pre-trained DNN that precisely aligned the nonlinear misalignments. To correct the OR-PAM image obtained by misaligned bidirectional scanning, we adapted a generative adversarial network (GAN) with U-Net^39–41^. In the GAN's learning method, two networks called the generator and discriminator are trained simultaneously and compete with one another^40^. As training advances, the generator learns to synthesize images that are more realistic, and the discriminator is trained to better distinguish the synthesized image from the real object. We built a generator based on Fully Dense U-Net (FD-U-Net), which performed well for PA imaging reconstruction (Fig. 2a and Supplementary Fig. S2)^27,42^. Our generator had four major modifications from the conventional FD U-Net: (1) multiscale input, (2) downsampling method, (3) upsampling method, and (4) number of downsampling and upsampling. In the first improvement, we downsampled the input images with median pooling and then adjusted the number of channels using a convolution layer with a kernel size of 1 × 1. Subsequently, the downsampled images were fed as inputs to the corresponding decoding blocks. We modified the network in this way to highlight the features of intersecting misalignment in the cross-sectional B-scans of the bidirectionally obtained image and to provide the network with various degrees of misalignment distribution for microvessels. Accordingly, we called our network “multiscale FD U-Net,” or MS-FD-U-Net. For the downsampling method, we adopted a convolutional layer with a stride of two, which could provide learned operations^43^. The third modification to the upsampling method was to replace the transposed convolution with a pixel shuffle operation in the expansion layer to remove unwanted checkerboard artifacts caused by the transposed convolution^44^. The fourth modification was to reduce the number of downsampling and upsampling blocks from four to three, which allowed us to achieve stable learning and better performance. Our generator contained approximately 7.6 million trainable parameters. For the discriminator, we utilized the previously reported discriminator, which comprised five convolutional layers linked in sequence. The network contained roughly 1.6 million learnable parameters (Fig. 2b)^37^.

We created a dataset consisting of paired images by imaging one region twice with a different number of pixels along the y-axis. After converting the acquired data to maximum amplitude projection (MAP) images, preprocessing was applied to roughly correct the distortion caused by angular scanning (Supplementary Fig. S3). Finally, all images were cropped to 192 pixels × 192 pixels. The bidirectional image was fed into the generator as the input, and the unidirectional image served as the ground truth. Especially, in this study, we acquired a total of 1154 OR-PAM paired images to train the network with various types of blood vessels and misalignment. The data were randomly divided into training set, validation set, and test set (Supplementary Table S1). The structural similarity (SSIM) metric was computed at each epoch for a validation set of 93 OR-PAM MAP images to store a checkpoint when training the network^45^. After 200 network training epochs, the trained network was evaluated using the test set.

### Qualitative and quantitative performance benchmark

**Figure 3:**
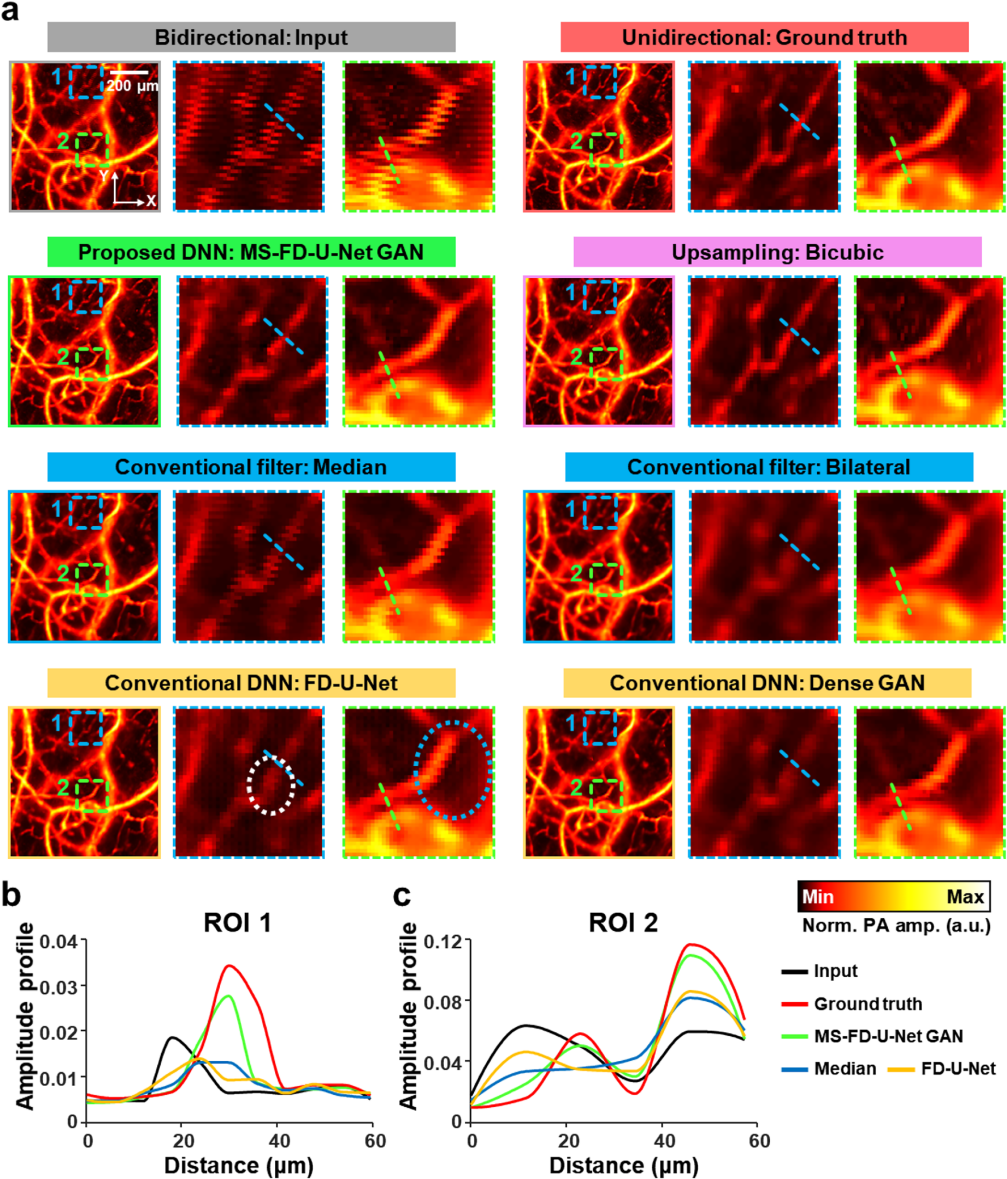
Performance comparison of the MS-FD-U-Net with other existing methods. (**a**) OR-PAM images reconstructed with bidirectionally (marked as Input) and unidirectionally (marked as Ground truth) acquired data, the MS-FD-U-Net GAN, an upsampling method (i.e., bicubic interpolation), conventional filtering methods (i.e., bilateral and median filtering), and other DNNs (i.e., Dense GAN and FD-U-Net). PA amplitude profiles from the regions highlighted by the (**b**) blue and (**c**) green dashed lines in (**a**), respectively. The graphs display the profiles in the images of Input, Ground truth, MS-FD-U-Net GAN, FD-U-Net and median filtering. The graphs for other methods (i.e., bicubic, bilateral filtering and dense GAN) are displayed in Supplementary Fig. S4.

To emphasize the advantages of our DNNs, we conducted a comparative study with conventional image enhancement filters (i.e., bicubic, bilateral and median filters) and previously reported DNN-based reconstruction methods (i.e., Dense GAN^36^ and FD U-Net^27^) (Fig. 3 and Table 1). The DNN-based reconstruction methods were trained and evaluated using the same training, validation, and test sets to ensure a fair comparison. When evaluating the filters, the same test set was used as in the evaluation of DNNs. Representative images and enlarged areas of the test set are shown in Fig. 3a. The MS-FD-U-Net results are superior in terms of perceptual similarity to the ground truth when compared to other methods. The amplitude profile graphs from the regions indicated by the dotted lines in Fig. 3a also demonstrate that our network most accurately restored the misaligned areas to the ground truth (Fig. 3b,c and Supplementary Fig. S4). It is worth noting that our major improvements (i.e., multiscale input and pixel-shuffle upsampling) in MS-FD-U-Net play a significant role in network performance. Misalignments in some blood vessels, highlighted by the white dotted circle in Fig. 3a, were not corrected in the results of the conventional FD-U-Net. By contrast, all misaligned vessels were properly restored in the image from our network. As a result of multiscale input, the network's learning can consider a wide range of misalignments. Additionally, by employing the pixel shuffle upsampling method, our network can synthesize more detailed parts of the blood vessels than the conventional FD-U-Net without unwanted checkboard artifacts, as shown in the region indicated by the blue dotted circle in Fig. 3a. The superiority of our network become apparent when the evaluation metrics are quantitatively compared (Table 1). MS-FD-U-Net consistently outperforms other enhancement methods. The results demonstrate that our MS-FD-U-Net is superior to existing methods in terms of image enhancement (i.e., structural similarity, SSIM^45^; multiscale SSIM, MS-SSIM^46^; peak SNR, PSNR; mean absolute error, MAE; and mean square error, MSE)^47^. We additionally introduced the blur absolute difference (BAD), which is calculated as the absolute difference of the blur metric (Supplementary Materials and Methods), to assess the blur quality of the corrected images compared to the ground truth^48^. In particular, our network yielded an exceptionally good BAD score. The inference time of our method was measured to be 12 ± 1 ms for the test set including 231 OR-PAM images. Because this time is negligible compared to the data acquisition time of 0.6 s, our method can be considered to accelerate the imaging time by approximately twofold.

**Table 1:** Statistical metrics (Mean ± SD) to compare the performances with test set.

		Upsampling	Conventional filter		Conventional DNN		Multi-scale sub-pixel FD-U-Net GAN
Metrics	Input	Bicubic	Bilateral	Median	Dense GAN	FD-U-Net	
SSIM	0.932 ± 0.055	0.912 ± 0.054	0.940 ± 0.050	0.939 ± 0.052	0.945 ± 0.049	0.942 ± 0.052	**0.948 ± 0.044**
MS-SSIM	0.948 ± 0.048	0.929 ± 0.049	0.953 ± 0.044	0.952 ± 0.046	0.956 ± 0.043	0.954 ± 0.046	**0.959 ± 0.039**
PSNR (dB)	38.11 ± 4.98	36.13 ± 4.54	38.77 ± 5.08	38.65 ± 5.14	38.64 ± 5.28	39.02 ± 5.28	**39.34 ± 4.68**
MAE (× 10^−3^)	7.85 ± 6.21	9.75 ± 6.70	7.52 ± 6.02	7.50 ± 6.15	7.14 ± 5.75	7.36 ± 6.04	**6.72 ± 5.11**
MSE (× 10^−4^)	3.55 ± 7.78	4.77 ± 8.86	3.22 ± 7.39	3.34 ± 7.64	3.30 ± 7.06	3.19 ± 7.41	**2.56 ± 6.27**
BAD (× 10^−2^)	2.34 ± 2.04	11.49 ± 3.57	15.88 ± 3.52	5.53 ± 2.32	10.11 ± 3.54	5.49 ± 2.76	**1.20 ± 0.94**

*SSIM* structural similarity, *MS-SSIM* multi-scale structural similarity, *PSNR* peak signal-to-noise ratio, *MAE* mean absolute error, *MSE* mean square error, and *BAD* blur absolute difference.

The top scores are highlighted in bold.

### Extrapolation study with 3D OR-PAM images

**Figure 4:**
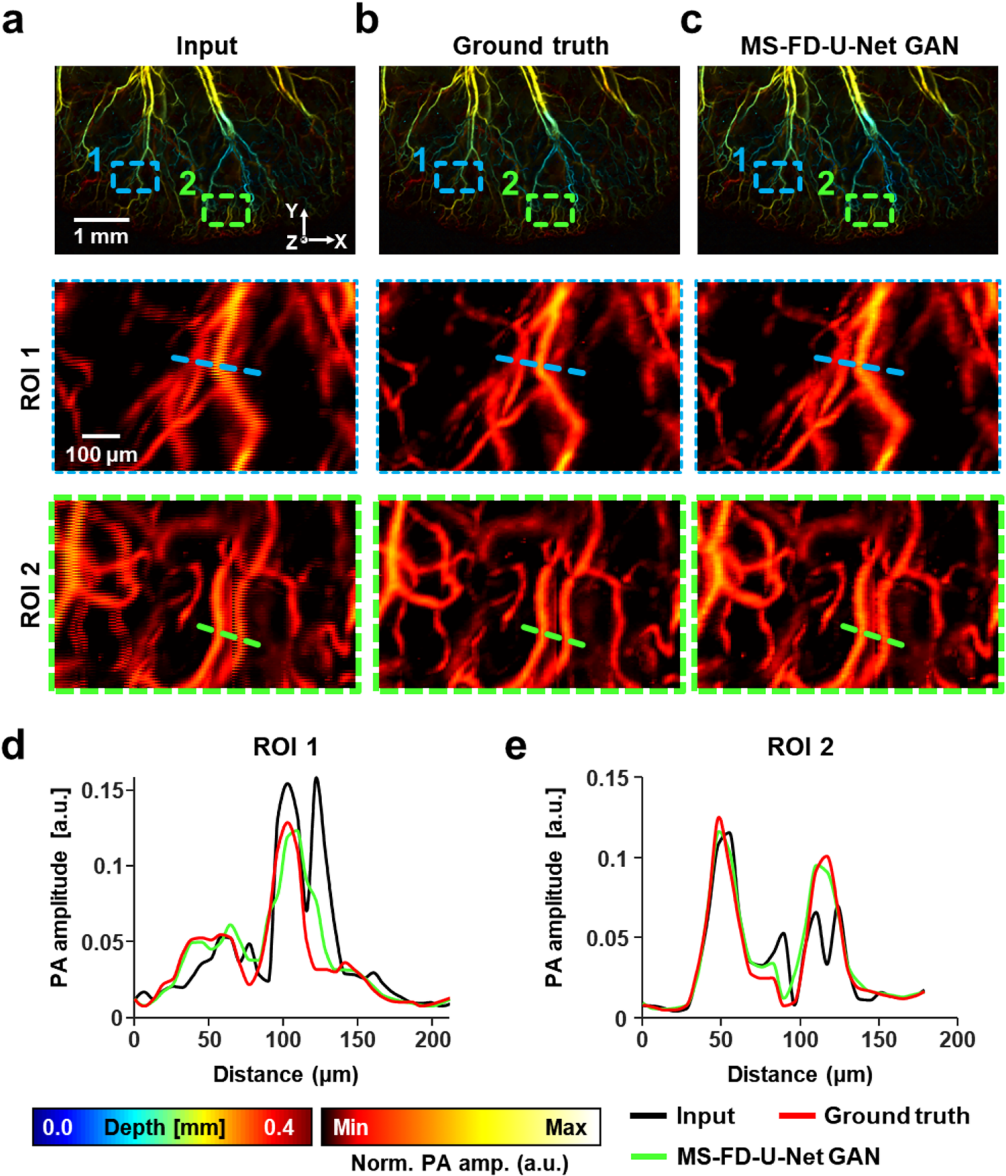
Extrapolation application of MS-FD-U-Net. (**a**–**c**) Depth-encoded PA images of a mouse’s ear reconstructed with (**a**) bidirectionally (marked as Input) and (**b**) unidirectionally (marked as Ground truth) acquired data, (**c**) the MS-FD-U-Net GAN. Close-up PA MAP views of ROI 1 and 2 in the regions indicated by the blue and green boxes are presented, respectively. PA amplitude profiles indicated by the dotted lines in (**d**) ROI 1 and (**e**) ROI 2, respectively. MAP, maximum amplitude projection; ROI, region of interest; and Norm. PA amp., normalized PA amplitude.

To demonstrate the extrapolation ability, we applied our pre-trained network to reconstruct 3D OR-PAM images acquired with a different field of view (FOV) to the training set (Fig. 4)^12^. We began the extrapolation study by acquiring 3D wide-FOV OR-PAM images with bidirectional raster scanning at a B-scan speed of 400 Hz as the input of the MS-FD-U-Net GAN (Fig. 4a) and an unidirectional raster scan at a B-scan speed of 200 Hz as the ground truth (Fig. 4b). The images, including 6 segmented images, has a FOV of 5.4 mm × 3.4 mm × 0.8 mm along the x-, y- and z-axes, respectively. The FOV of the training set was 1.1 mm × 1 mm, while the FOV of each segment image was 1.7 × 4 mm along the x- and y-axes, respectively. Because our pre-trained network included 2D operations, we applied the network to cross-sectioned *enface* images of the 3D OR-PAM images along the z-axis (Supplementary Figs. S5 and S6). After using our pre-trained network to correct all the sectioned *enface* images, the improved 2D images were reassembled into 3D images (Fig. 4c). All images are represented using a depth-encoded map that provides 3D structural information. To facilitate image quality comparison, enlarged images are displayed as MAP images. By comparing the enlarged images, it is confirmed that the pre-trained network correctly mended the misaligned pattern not only for the 3D image, but also for images of different scanning lengths from the training set (Fig. 4a–c). The amplitude profile graphs of the locations denoted by the dotted lines in the enlarged regions demonstrate that the images were improved (Fig. 4d,e). In the input profile, one blood vessel is divided into two by the misaligned pattern. However, the images improved by our network describe blood vessels similar to the ground truth. The evaluation metrics also prove that our network improved the bidirectionally obtained OR-PAM images (Supplementary Table S2). These results validate our network's extrapolation capability to synthesize volumetric OR-PAM images.

## Discussion

We introduced a deep-learning-based PA imaging method to correct the distortion caused by bidirectional raster scanning. In general, to reconstruct OR-PAM images, the OR-PAM system that acquires images via raster scanning uses data obtained with unidirectional scanning to avoid the misalignment that occurs with bidirectional scanning. Although unidirectional scanning can produce high-quality images, this method sacrifices the effective B-scan rate. The implemented DNN-based imaging framework perceptually creates superior OR-PAM images from misaligned OR-PAM images using bidirectional scanning. Our framework reduces the imaging time by half by eliminating wasted scanning, resulting in double the speed of the effective B-scan rate. This result demonstrates that our technology can double the effective B-scan rate of raster-scan-based OR-PAM systems without requiring any hardware updates.

To successfully implement our framework in detail, we introduced the following strategies: image pairs derived from real-world imaging scenarios, large datasets, and modification of the generator network. First, to train a high-performance DNN that can be used in practice, image pairs were obtained in real-world imaging scenarios that were not generated by simulation or image processing. OR-PAM images from unidirectional and bidirectional scans were used as the ground truth and input, respectively. In a previous undersampling study, image pairs were acquired through artificial downsampling processing^27^. Although good results were obtained with the image pairs, there is a difference between the undersampled and fully sampled scanning paths; therefore, the artificial downsampling method does not perfectly depict the actual imaging. In this study, we were able to fully reflect the actual imaging path differences by training the DNNs with image pairs obtained using unidirectional and bidirectional scans. Second, we used over 1000 OR-PAM MAP images obtained at two imaging rates for training, allowing the network to learn various types of distortion. Together with this, the network improvement for multiscale inputs enabled our network to achieve extrapolation capability, which allowed the DNNs to improve various bidirectional scanning of OR-PAM images.

The scope of future research includes further applying the proposed pre-trained network to other biomedical imaging modalities (i.e., OCT and ultrasound microscopy) to prove its extrapolation ability. Other modalities also experienced misalignment during fast bidirectional raster scanning, resulting in unoptimized scanning times^13,19,49–51^. Therefore, we aim to prove the general ability of our DNN framework by correcting misaligned images from various biomedical imaging modalities.

By improving the misaligned image gathered with the bidirectional raster scan, our deep learning framework enhances the potential of the existing OR-PAM. This DNN-based OR-PAM imaging provides 3D OR-PAM images twice as fast as conventional methods without requiring hardware updates, allowing for the investigation of phenomena such as instantaneous drug responses that were not recorded by conventional imaging. In particular, our framework significantly reduces the number of laser pulses irradiated on the sample and the total imaging time, thereby alleviating the burden on the subject during imaging. Owing to this advantage, it has the potential to be used for monitoring and diagnosing diseases associated with sensitive skin, such as melanomas, fungal infections, and warts. With the doubling of the imaging speed, the ability to monitor brain hemodynamics and neuronal activity using OR-PAM is also enhanced.

## Materials and methods

### Volumetric OR-PAM data acquisition and preprocessing

To obtain a dataset for training DNNs, our previously reported OR-PAM system with a water-immersible galvanometer (OptichoM, Opticho, South Korea) was used in the current work^37^. It employed a fast nanopulse laser system with a maximum PRR of 600 kHz (VPFL-G-10, Spectra-Physics, USA) to induce PA waves. A galvanometer scanner (GVS001, Thorlabs, USA) and a linear motorized stage (L-406, Physik Instrumente LTD, UK) were used to scan the samples using raster scanning. The raster scanning pattern was formed by simultaneously performing slow scanning of the linear motor stage and rapid angular scanning of the galvanometer scanner. The generated PA waves were measured using a ring-shaped ultrasonic transducer with an inner diameter of 3.1 mm, an outer diameter of 15 mm and a focal length of 21 mm.

The training and test set consisted of OR-PAM images obtained from mice’s ears with a FOV of 1.1 mm × 1 mm, and the number of pixels being 250 pixels along the x-axis and 500 or 250 pixels along the y-axis. We prepared paired images by adjusting the step size along the y-axis to 2 μm (for unidirectional scanning) and 4 μm (for bidirectional scanning). We acquired a total of 1154 OR-PAM paired images and randomly divided them into training and test sets at a ratio of 8:2 (Supplementary Table S1). The training set was then randomly split into training and validation set at a ratio of 9:1, to be used for training. For the extrapolation study, we prepared wide-FOV OR-PAM images including six segmented images with an FOV of 1.7 mm × 4 mm × 0.8 mm along the x-, y-, and z-axes, respectively. The step sizes were 6.8 μm × 5 × 3 μm along the x-, y-, and z-axes, respectively.

### Deep neural network

Our framework consisted of two DNNs: a generator and a discriminator. By customizing the pix2pix architecture^41^ for image-to-image transition, we developed our DNN architecture of the generator (Fig. 2). Generator *G* was revised from U-net. The generator network was composed of four downsampling (in an encoding network) and four upsampling (in a decoding network) convolutional layers. Five convolutional blocks were serially linked to form the discriminator *D*. The leaky rectified linear unit (LReLU)^52^ function were used as the activation function and a sigmoid activation function was used as the output convolutional layer.

We used the GAN framework to train the generator and discriminator alternately to address the adversarial min–max problem and improve results:

1 $$\mathop {\min }\limits_{G} \mathop {\max }\limits_{D} E_{{y\sim P_{data\left( y \right)} }} \left[ {\log D\left( y \right)} \right] + E_{{x\sim P_{data\left( x \right)} }} \left[ {\log \left( {1 - D\left( {G\left( x \right)} \right)} \right)} \right],$$

where *x* is a bidirectional scanning OR-PAM image as the input of the DNNs and *y* is the corresponding unidirectional scanning OR-PAM image used as the ground truth. We designed an adversarial loss combined with two regularization terms: the mean absolute error (MAE) and structural similarity (SSIM) index:

2 $$L^{Adv} = \lambda \times \left( { - \log D\left( {G\left( x \right)} \right) + \nu \times \frac{1}{N}\sum \left| {y - G\left( x \right)} \right| + \left( {1 - \lambda - \nu } \right) \times \left( {1 - SSIM\left( {y, G\left( x \right)} \right)} \right),} \right.$$

where *N* the total number of pixels in the PA image. Rather than employing MSE, which produced unsatisfactory outcomes in image-to-image transition works, we integrated adversarial loss with MAE^40,47^. The He normal initialization method was used to initialize all of the trainable parameters^52^. The parameters were optimized by the Adam optimizer^53^. In addition, to prevent overfitting network parameters, an L2 regularization technique was used^54^. With the validation set, we assessed the SSIM metrics during training to define the model checkpoints. The Bayesian Optimization Hyperband (BOHB) was used to improve hyperparameters such as loss function coefficients, learning rates, and batch sizes with a reduction factor of 2, a maximum time unit per trial of 20, and trials of 200^55^. The hyperparameters are summarized in Supplementary Table S3. In the case of a dense network, the number of blocks and convolutional layers within a block were additionally set as hyperparameters. After determining the optimal parameters for each deep-learning model, the network was trained for up to 200 epochs. All the DNNs were built with Python 3.8.3 and PyTorch, and training was performed using Intel®Core™ i9-10900X CPU and NVIDIA RTX 3090 GPUs.

### Photoacoustic imaging of animals in vivo

All animal treatments were performed in accordance with the National Institutes of Health Guide for the Care and Use of Experimental Animals, with authorization from the Institutional Animal Care and Use Committee of Pohang University of Science and Technology (POSTECH). This study was also performed in accordance with ARRIVE guidelines. Female BALB/c mice aged 3–8 weeks were anesthetized with 4% isoflurane gas at a flow rate of 1.0 L/min and kept warm using a silicone heating pad placed beneath the mice during photoacoustic imaging. A 532 nm optical beam with a pulse energy of 10 mJ/cm^2^ was utilized in the imaging studies, which was less than the ANSI safety limit of 20 mJ/cm^2^. Prior to imaging, the hair was cleaned using a depilatory cream to optimize the transmission of light to the target. To reduce PA waves reflection between the target and the transducer, an impedance matching gel was placed between the mouse's ear and the polyvinyl chloride membrane of the water tank. A total of 30 mice were used in this study.

## Supplementary Information

Supplementary Information.

## Data Availability

All data are available within the Article and Supplementary Files or available from the authors upon request.
