# Supplementary material for: Deep learning alignment of bidirectional raster scanning in high speed photoacoustic microscopy
Source: Sci Rep. 2022 Sep 28;12:16238. doi: 10.1038/s41598-022-20378-2 (PMC9519743; doi:10.1038/s41598-022-20378-2)
Supplement: Supplementary file 1 — Supplementary Information. [file 41598_2022_20378_MOESM1_ESM.docx]

Supplementary Materials for

**Deep Learning Alignment of Bidirectional Raster Scanning in High Speed Photoacoustic Microscopy**

Jongbeom Kim, Dongyoon Lee, Hyokyung Lim, Hyekyeong Yang, Jaewoo Kim, Jeesu Kim, Yeonggeun Kim, Hyung Ham Kim, and Chulhong Kim^*^

^*^Corresponding author. Email: chulhong@postech.edu

**This file includes:**

**Supplementary Materials and Methods**

**Supplementary Fig. S1.** Spatial resolution of optical-resolution photoacoustic microscopy (OR-PAM) system.

**Supplementary Fig. S2.** Visual representation of conventional FD-U-Net architecture.

**Supplementary Fig. S3.** Illustration of the preprocessing.

**Supplementary Fig. S4.** Performance comparison of MS-FD-U-Net GAN, FD-U-Net, and median filtering on small vessels.

**Supplementary Fig. S5.** Extrapolation study process with 3D OR-PAM.

**Supplementary Fig. S6.** Representative corrected sectioned *enface* images along the z-axis.

**Supplementary Table S1.** Number of OR-PAM images of in vivo mouse ears used in this study.

**Supplementary Table S2.** Statistical metrics (mean ± SD) were used to compare the performances with wide FOV images.

Supplementary Table S3. Training setting of MS-FD-U-Net GAN.

**Supplementary Materials and Methods:**

**Blur absolute difference**

Blur absolute difference (BAD) metric, which is based on the no-reference perceptual blur metric previously reported by Crété-Roffet^1^, is defined by as follows:

$BAD\left( x, y \right)=\left| blur\left( x \right)-blur(y) \right|$*,*

where *x* is an input image and *y* is a ground truth image. The blur metric^1^ is calculated as follows:

$h_{v}=\frac{1}{9}\times\left[ 1 1 1 1 1 1 1 1 1 \right]$ $h_{h}=transpose\left( h_{v} \right)$

$B_{v}=h_{v}*F$ $B_{h}=h_{h}*F$,

where *F* is a gray scale input image with a pixel size of m × n. First, the variations of the neighboring pixels are studied as follows:

$${D\_F}_{\boldsymbol{v}}\left( i,j \right)\boldsymbol{=}Abs\mathbf{(}F\left( i,j \right)-F\left( i-1,j \right)) for i=1 to m-1, j=0 to n-1$$

$${D\_F}_{h}\left( i,j \right)\boldsymbol{=}Abs\mathbf{(}F\left( i,j \right)-F\left( i-1,j \right)) for j=1 to n-1, i=0 to m-1$$

$${D\_B}_{\boldsymbol{v}}\left( i,j \right)\boldsymbol{=}Abs\mathbf{(}B_{v}\left( i,j \right)-B_{v}\left( i-1,j \right)) for i=1 to m-1, j=0 to n-1$$

$${D\_B}_{h}\left( i,j \right)\boldsymbol{=}Abs\mathbf{(}B_{h}\left( i,j \right)-B_{h}\left( i-1,j \right)) for j=1 to n-1, i=0 to m-1$$

$$V_{v}=Max(0,D\_F_{v}(i,j)-D\_B_{v}(i,j)) for i=1 to m-1, j=1 to n-1$$

$V_{h}=Max(0,D\_F_{h}(i,j)-D\_B_{h}(i,j)) for i=1 to m-1, j=1 to n-1$.

Then, the sum of the coefficients to compare the variations from the gray scale input image as follows,

$S\_F_{v}=\sum_{i,j=1}^{m-1,n-1} D\_F_{v}(i,j)$ $S\_F_{h}=\sum_{i,j=1}^{m-1,n-1} D\_F_{h}(i,j)$

$S\_V_{v}=\sum_{i,j=1}^{m-1,n-1} D\_V_{v}(i,j)$ $S\_V_{h}=\sum_{i,j=1}^{m-1,n-1} D\_V_{h}(i,j)$

Finally, after the results are normalized, the blur is selected as the maximum among the vertical one and the horizontal one,

$b\_F_{v}=\frac{S\_F_{v}-S\_V_{v}}{S\_F_{v}}$ $b\_F_{h}=\frac{S\_F_{h}-S\_V_{h}}{S\_F_{h}}$

$Blur=Max(b\_F_{v}, b\_F_{h})$.

**
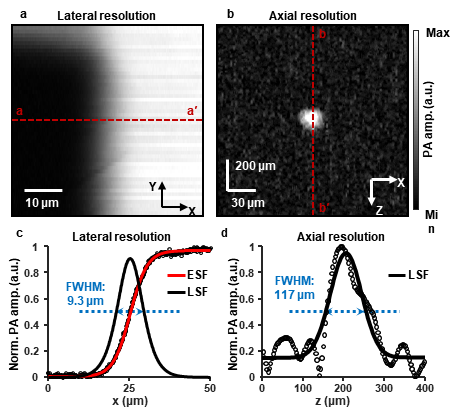
Supplementary Figures:**

**Supplementary Fig. S1.** **Spatial resolution of optical-resolution photoacoustic microscopy (OR-PAM) system.** (**a**) OR-PAM MAP image of a patterned microstructure. (**b**) Cross-sectional OR-PAM B-mode image of a carbon fiber. (**c**) Fitted ESF and LSF of OR-PAM data indicated by the line a-a′ in (**a**)*.* LSF is calculated from the first derivative of the ESF. The lateral resolution was measured as the FWHM of the LSF. (**d**) Fitted LSF of OR-PAM data indicated by the line b-b′ in (**b**). Axial resolution was measured as the FWHM of the LSF. PA, photoacoustic; ESF, edge spread function; LSF, line spread function; MAP, maximum amplitude projection; FWHM, full width at half maximum.

**
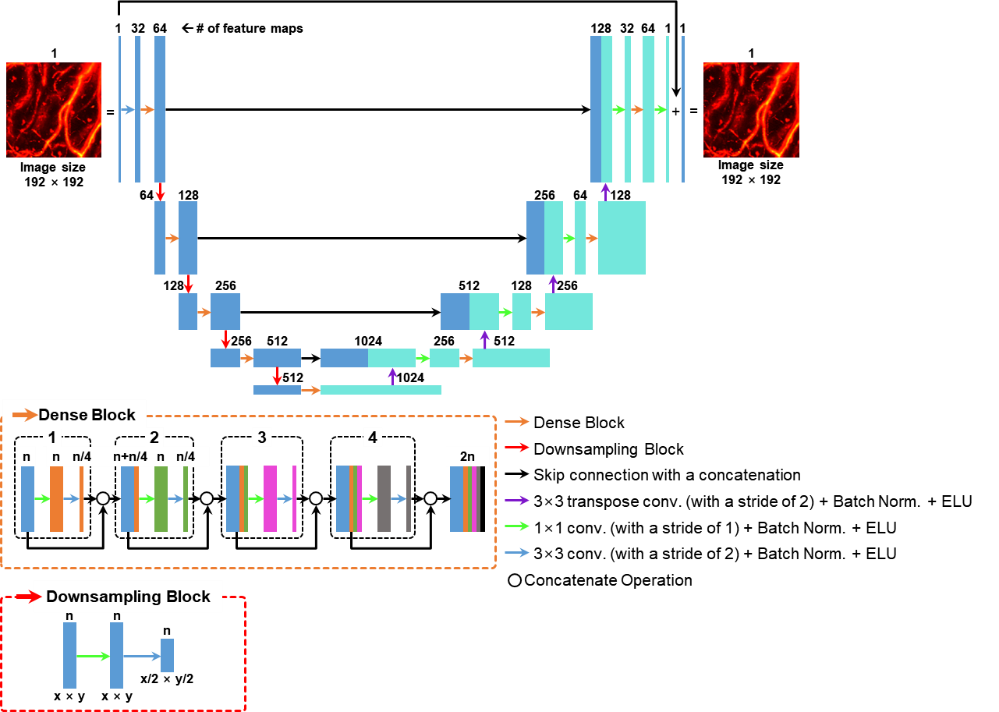

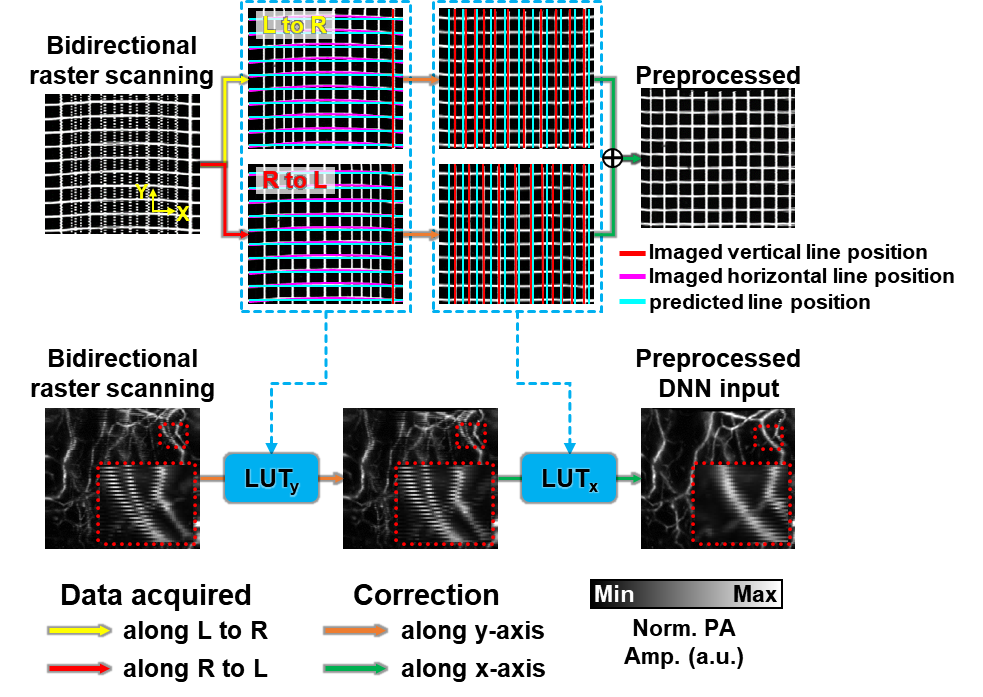
**

Supplementary Fig. S2. Visual representation of conventional FD-U-Net architecture.

**Supplementary Fig. S3. Illustration of the preprocessing.** Distortion information of the parabolic scanning pattern is measured from the grid images. The bidirectional image is corrected after divided into two images depending on the scanning direction. The red and purple lines highlight vertical and horizontal lines on the PA image, respectively. The cyan line indicates the expected actual position of the line. The lookup table in the y-axis direction is calculated as the difference between the purple line and the cyan line, and the lookup table in the x-axis direction is calculated as the difference between the red line and the cyan line. The lookup tables obtained while correcting the grid image are applied to the OR-PAM microvascular image. LUT, lookup table; L, left; R, right; and Norm. PA. Amp., normalized photoacoustic amplitude.

**Supplementary Fig. S4.** **Performance comparison of MS-FD-U-Net GAN, FD-U-Net, and median filtering on small vessels.** (**a**), (**b**) Profiles of the PA amplitude indicated by the (**c**) blue and (**d**) green dashed lines in Fig. 3, respectively. The graphs display the profiles in the images of Input, Ground truth, MS-FD-U-Net GAN, Dense GAN, bicubic interpolation, and bilateral filter.


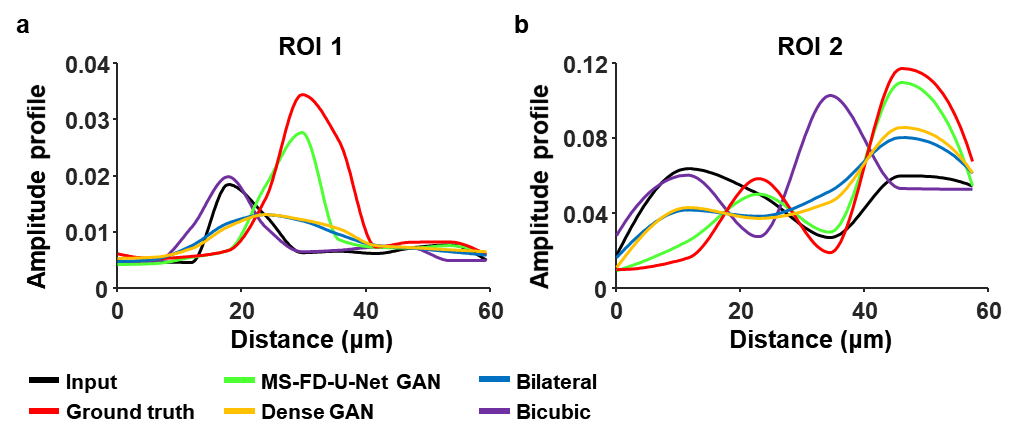


**Supplementary Fig. S5.** **Extrapolation study process with 3D OR-PAM.** The area highlighted by white dotted circles demonstrates that misalignment has been corrected via our DNN. DNN, deep neural network.


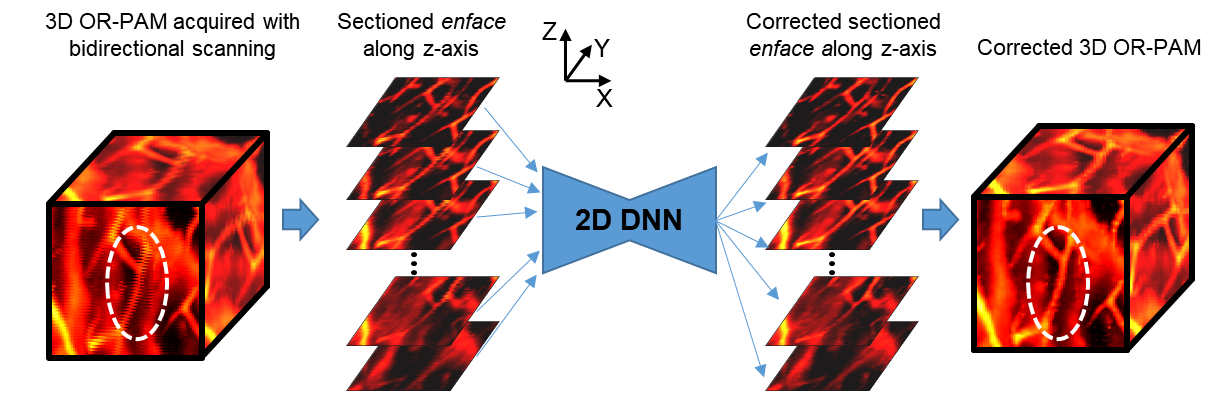


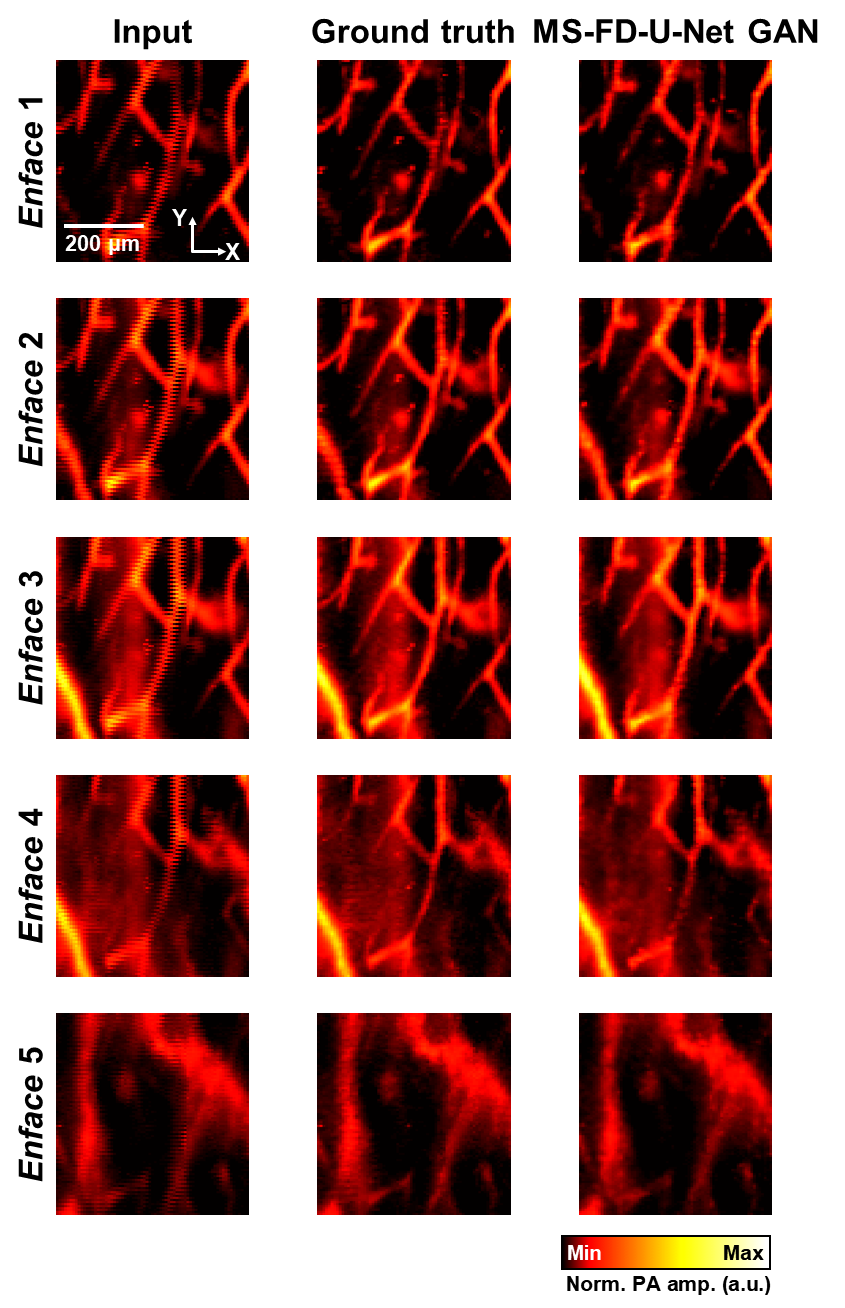
Supplementary Fig. S6. Representative corrected sectioned *enface* images along the z-axis.

| **Training set** | | **Test set** |
| --- | --- | --- |
| 923 (80%) | | 231 (20%) |
| **Training set** | **Validation set** | **Test set** |
| 830 (72%) | 93 (8%) | 231 (20%) |

Supplementary Table S1. Number of OR-PAM images of *in vivo* mouse ears used in this study.

Supplementary Table S2. Statistical metrics (mean ± SD) were used to compare the performances with wide FOV images.

|  |  | **Multi-scale  FD-U-Net GAN** |
| --- | --- | --- |
| **Metrics** | **Input** |  |
| **SSIM** | 0.993±0.002 | **0.994±0.002** |
| **MS-SSIM** | 0.998±0.001 | **0.998±0.001** |
| **PSNR (dB)** | 50.22±2.52 | **50.62±2.50** |
| **MAE (×10^-3^)** | 1.17±0.22 | **1.12±0.20** |
| **MSE (×10^-5^)** | 1.09±0.60 | **0.99±0.50** |
| **BAD (×10^-2^)** | 1.94±1.18 | **1.78±1.12** |

SSIM, structural similarity; MS-SSIM, multi-scale structural similarity; PSNR, peak signal-to-noise ratio; MAE, mean absolute error; MSE, mean square error; and BAD, blur absolute difference.

Supplementary Table S3. Training setting of MS-FD-U-Net GAN.

| **Training settings** | | | | | | |
| --- | --- | --- | --- | --- | --- | --- |
| Module | Learning rate | Beta1 | Beta2 | Batch size | Adversarial loss coefficient | L1 coefficient |
| Generator | 0.0003847937179628929 | 0.5 | 0.5 | 4 | 0. 030525062069017193 | 0.38505600988263033 |
| Discriminator | 0.00011228967280528144 | 0.5 | 0.5 |  | - | - |

**Reference**

1 Crete, F., Dolmiere, T., Ladret, P. & Nicolas, M. The blur effect: perception and estimation with a new no-reference perceptual blur metric. in *Human vision and electronic imaging XII.* 64920I (International Society for Optics and Photonics).
